# Cardiovascular disease treatment among patients with severe mental illness: a data linkage study between primary and secondary care

**DOI:** 10.3399/bjgp16X685189

**Published:** 2016-04-26

**Authors:** Charlotte Woodhead, Mark Ashworth, Matthew Broadbent, Felicity Callard, Matthew Hotopf, Peter Schofield, Murat Soncul, Robert J Stewart, Max J Henderson

**Affiliations:** King’s College London, Institute of Psychiatry, Psychology and Neuroscience, London.; King’s College London, Institute of Psychiatry, Psychology and Neuroscience, London.; Head of information governance, South London and Maudsley NHS Foundation Trust, London.; Reader in social science for medical humanities, Durham University, Centre for Medical Humanities, Durham.; King’s College London, Institute of Psychiatry, Psychology and Neuroscience, London.; King’s College London, Institute of Psychiatry, Psychology and Neuroscience, London.; Head of information governance, South London and Maudsley NHS Foundation Trust, London.; King’s College London, Institute of Psychiatry, Psychology and Neuroscience, London.; King’s College London, Institute of Psychiatry, Psychology and Neuroscience, London.

**Keywords:** cardiovascular diseases, data linkage, health inequalities, primary health care, psychoses

## Abstract

**Background:**

Suboptimal treatment of cardiovascular diseases (CVD) among patients with severe mental illness (SMI) may contribute to physical health disparities.

**Aim:**

To identify SMI characteristics associated with meeting CVD treatment and prevention guidelines.

**Design and setting:**

Population-based electronic health record database linkage between primary care and the sole provider of secondary mental health care services in south east London, UK.

**Method:**

Cardiovascular disease prevalence, risk factor recording, and Quality and Outcomes Framework (QOF) clinical target achievement were compared among 4056 primary care patients with SMI whose records were linked to secondary healthcare records and 270 669 patients without SMI who were not known to secondary care psychiatric services, using multivariate logistic regression modelling. Data available from secondary care records were then used to identify SMI characteristics associated with QOF clinical target achievement.

**Results:**

Patients with SMI and with coronary heart disease and heart failure experienced reduced prescribing of beta blockers and angiotensin-converting enzyme inhibitor/angiotensin receptor blockers (ACEI/ARB). A diagnosis of schizophrenia, being identified with any indicator of risk or illness severity, and being prescribed with depot injectable antipsychotic medication was associated with the lowest likelihood of prescribing.

**Conclusion:**

Linking primary and secondary care data allows the identification of patients with SMI most at risk of undertreatment for physical health problems.

## INTRODUCTION

Patients with severe mental illness (SMI), including schizophrenia, bipolar affective disorder, and schizoaffective disorder or other non-organic psychoses, experience lower life expectancy than the general population.^1^–^4^ This is largely attributed to common physical disorders, particularly cardiovascular diseases (CVDs).^2^,^3^,^5^,^6^

Excess mortality linked to CVDs is attributed to several factors, including elevated risk factors such as smoking; side effects of pharmacological treatment; diagnostic overshadowing; and, suboptimal management of comorbid physical conditions.^7^–^14^ Previous studies have been unable to investigate associations for varying SMI-related characteristics as data on physical health and clinical management sit mainly within primary care, whereas mental health condition and management records are mainly stored in secondary care.

This study uses London borough population-based data from a linkage of primary and secondary mental healthcare records to: compare CVD prevalence, risk factor recording and treatment for established CVD, and primary care consultation frequency by SMI status; examine whether SMI characteristics are differentially associated with CVD prevalence and treatment; and assess the impact of adjustments for consultation frequency.

## METHOD

### Setting and data sources

Lambeth is a diverse borough in south east London, with a greater proportion of black Caribbean and black African residents but fewer South Asian residents than other areas,^15^ and is more deprived than England as a whole.^16^ Pseudonymised primary care data were extracted on 31 March 2013 from computerised medical records of all except one GP practice (*n* = 48) within Lambeth, as part of Lambeth DataNet (LDN) covering a population of 366 317 registered patients.

This was a cross-sectional extract of LDN, but for some records (for example blood pressure [BP]), information on all measures recorded from 31 January 2012 to 31 October 2013 were collected to determine whether Quality and Outcomes Framework (QOF)^17^ clinical targets had been met. Secondary care data came from the Case Register Interactive Search (CRIS),^18^ an application allowing researchers access to pseudonymised electronic health record (EHR) data from the South London and Maudsley NHS Foundation Trust (SLaM). CRIS provides searchable access to de-identified text (unstructured data) from the clinical record.

### Data linkage

Data were linked and stored by the Clinical Data Linkage Service (CDLS), which provides a safe haven environment with strict governance arrangements. Data were linked using encrypted NHS numbers, which were subsequently removed and destroyed, fully anonymising the linked dataset.

**How this fits in**

Patients with severe mental illness (SMI) experience lower life expectancy than the general population. Suboptimal treatment of cardiovascular diseases has been identified as a potential contributory factor. This study found that patients with SMI in south east London are underprescribed beta blockers and angiotensin converting enzyme inhibitors/angiotensin receptor blockers as secondary prevention after coronary heart disease and heart failure. This may help clinicians to identify patients at greatest risk of suboptimal treatment.

### Measures

#### Lambeth DataNet (LDN)

Data were extracted on sex, year of birth, ethnic group, and 2011-defined lower super output area (LSOA). LSOA data were used to estimate deprivation on the basis of patient area of residence using the Index of Multiple Deprivation (IMD-2010) and a conversion to 2011 LSOA values. GP clinical register data (lists established and maintained by practices of patients identified with particular clinical outcomes for QOF purposes) were collected for heart failure (HF), coronary heart disease (CHD), hypertension (HYP), and stroke/transient ischaemic attack (STIA). Data were also collected on CVD risk factor recording, for example BP; clinical values and dates; and, mean number of primary care consultations (including GP, nurse, face-to-face, and telephone) between 2010 and 2013. A binary variable was created to distinguish median or below and above median annual number of consultations.

#### Case Register Interactive Search (CRIS)

Diagnostic codes for any primary or secondary diagnosis of schizophrenia, bipolar affective disorder, and schizoaffective disorder or other non-organic psychoses were extracted. An indicator of SMI severity was created, coding patients with SMI as 1 if they ever had a record of: an inpatient stay, being treated under the Mental Health Act, difficulty managing their physical health, or contact with Assertive Outreach, Crisis or A&E liaison team (or 0 if they had not been recorded with any of these). Similarly, an indicator of risk coded patients with SMI as 1 or 0 to indicate if they had ever been identified under the ‘violence and aggression’ subscale of risk assessment with a history of violence, non-compliance, or forensic history. Lastly, binary indicators of antipsychotic medication prescription were extracted; including binary indicators of atypical, typical, and depot injectable medication.

### Statistical analyses

Pearson’s χ^2^ tests and logistic regression analyses were used to compare CVD prevalence, risk factor recording, QOF target achievement, and primary care consultation frequency by SMI status. Using linked data, comparisons by SMI status in CVD prevalence and prescribing were then examined by individual SMI characteristics. Logistic regression analyses were used to assess whether any differences in CVD prevalence or prescribing could be accounted for by adjustment for sociodemographic characteristics and consultation frequency. *P*-values, unadjusted and adjusted odds ratios (OR), and 95% confidence intervals (CI) are shown. The large number of statistical tests conducted meant that an α level of *P*<0.01 was used to determine statistical significance. All analyses were conducted using Stata (version 12).

## RESULTS

Data were obtained for LDN patients aged ≥16 years (*n* = 295 301); of these, 8.1% (*n* = 23 919) were linked to secondary mental healthcare records. Among those with linked records, *n* = 4056 (16.9%) were recorded with SMI by their GP in LDN. Analyses compared those with recorded SMI in primary care with linked secondary care records (*n* = 4056) to those not recorded with SMI in primary care or linked to secondary care (*n* = 270 669).

### Sociodemographics, CVD prevalence, and consultation frequency among patients with and without SMI

SMI status was associated with sex, age, ethnic group, deprivation, consultation frequency, and greater prevalence of CVDs (Table 1). In patients with an established CVD (data not shown) there were no longer associations between SMI status and sex, nor age among patients with CHD or STIA. SMI status was only associated with ethnic group and GP consultation rate among patients with HYP, and SMI status was no longer associated with deprivation among patients with any CVD condition.

**Table 1.** Sociodemographic characteristics and CVD prevalence by severe mental illness (SMI) status

	**Non-SMI (N = 270 669), *n* (%)**	**SMI (*N* = 4056), *n* (%)**	***P*-value**
**Sex^a^**			<0.001^b^
Female	137 353 (50.8)	1797 (44.3)	
Male	133 315 (49.3)	2259 (55.7)	

**Age group, years**			<0.001^b^
16–24	32 776 (12.1)	162 (4.0)	
25–34	88 062 (32.5)	678 (32.5)	
35–44	59 279 (21.9)	907 (22.4)	
45–54	42 839 (15.8)	1095 (27.0)	
55–64	23 734 (8.8)	624 (15.4)	
65–74	14 035 (5.2)	347 (8.6)	
≥75	9944 (3.7)	243 (6.0)	

**Ethnic group**			<0.001^b^
British/mixed	78 332 (35.0)	1124 (31.6)	
Irish	5253 (2.4)	104 (2.9)	
Indian/Pakistani/Bangladeshi/mixed	16 042 (7.2)	219 (6.2)	
Caribbean/mixed	21 401 (9.6)	840 (23.7)	
African/mixed	27 286 (12.2)	545 (15.3)	
Chinese/other	10 871 (4.9)	90 (2.5)	
Other white	54 080 (24.2)	373 (10.5)	
Other black	6262 (2.8)	188 (5.3)	
Other mixed	4254 (1.9)	69 (1.9)	

**Deprivation quintile**			<0.001^b^
1 Most deprived	47 162 (18.1)	1004 (25.0)	
2	54 656 (21.0)	918 (22.9)	
3	54 342 (20.9)	836 (20.8)	
4	57 149 (22.0)	713 (17.8)	
5 Least deprived	47 054 (18.1)	543 (13.5)	

**Consultations**			
Mean (SD)	4.7 (4.3)	9.4 (8.0)	
Median/below	123 501 (53.1)	813 (20.9)	<0.001^b^
Above median	109 286 (47.0)	3074 (79.1)	

**Cardiovascular diseases**			
Hypertension	28 010 (10.4)	762 (18.8)	<0.001^b^
Coronary heart disease	4109 (1.5)	97 (2.4)	<0.001^b^
Heart failure	1259 (0.5)	45 (1.1)	<0.001^b^
Stroke/transient ischaemic attack	2544 (0.9)	100 (2.5)	<0.001^b^

SD = standard deviation. SMI = severe mental illness. Patients with SMI are those known to both primary and secondary care, patients without SMI are those known only to primary care and not registered with a SMI. ‘Consultations’ refers to mean number of GP and nurse telephone, face-to-face and home primary care consultations per calendar year between 2010 and 2013.

a One patient recorded as sex ‘unknown’.

b *P* <*0.001.*

### Sociodemographic characteristics of SMI subgroups

The SMI characteristics extracted from secondary care data are shown in Table 2. Adjusting for all sociodemographic characteristics simultaneously (data not shown), being black African, black Caribbean, other black, and of a younger age were associated with indicators of risk and severity, and with receiving depot injectable antipsychotic medication; male sex was also associated with risk. Being black Caribbean and older was associated with receipt of typical antipsychotics, whereas younger age and being black African was associated with receipt of atypical antipsychotics. Relative to those with a diagnosis of schizophrenia, those diagnosed with bipolar disorder were younger, more likely to be identified as British/mixed British, female, and to consult primary care more frequently (*P* = 0.01). Those diagnosed with schizoaffective disorder/other non-organic psychoses were younger, more likely to be female, and to consult primary care less frequently relative to patients with schizophrenia (except where indicated, all *P*-values <0.001).

**Table 2.** Indicators of severity and risk identified from secondary care data among patients with severe mental illness

	***N* = 4056, *n* (%)**
**Diagnosis**	
Schizophrenia	1721 (53.6)
Bipolar affective disorder	716 (22.3)
Other non-organic psychoses	773 (24.1)

**Indicator of severity, ever:**	2147 (53.0)
Treated under Mental Health Act	1416 (34.9)
Inpatient	1927 (47.5)
Seen by crisis team	23 (0.6)
Seen by assertive outreach	11 (0.3)
A&E outpatient episode	445 (11.0)
Difficulty managing physical health	676 (16.7)

**Indicator of risk, ever:**	1751 (43.0)
History of non-compliance	1296 (32.0)
History of violence	1171 (28.9)
Forensic history	620 (15.3)

**Antipsychotics, ever:**	
Depot injectable	1112 (32.3)
Atypical	3255 (94.5)
Typical	1506 (43.7)

### CVD risk factor recording and QOF target achievement

CVD risk factor recording (for example BP) was, in general, high for patients with and without SMI (Table 3). Among those with established CVDs, patients with SMI were more likely to have a record of their alcohol intake. Among patients with HYP, SMI status was also associated with greater recording of body mass index and glycated haemoglobin levels. Patients with SMI with CHD were less likely to have a BP record, whereas those with STIA were less likely to have a record of BP and smoking status. CVD risk assessment (for example Framingham risk score) was significantly less common among patients with SMI. Despite significantly higher prevalence of CVDs in the SMI group overall, there was little or no difference in the prevalence of comorbid CVDs or diabetes by SMI status among those with established CVDs. Among patients with HYP, diabetes was significantly more common among patients with SMI than without. For most QOF targets, there was no significant difference between patients with SMI and patients without SMI. For patients with SMI as well as HF and CHD, a significant shortfall was observed in prescribing with ACE inhibitors or angiotensin receptor blockers (ACEIs/ARBs) and beta blockers.

**Table 3.** CVD risk factor recording and QOF CVD target achievement by serious mental illness (SMI) status and among patients with CVD conditions

**Risk factor recording**	**Heart failure**			**Coronary heart disease**			**Hypertension**			**Stroke/transient ischaemic attack**		
			
	**Non-SMI (*n* = 1259)**	**SMI (*n* = 45)**	***P*-value**	**Non-SMI (*n* = 4109)**	**SMI (*n* = 97)**	***P*-value**	**Non-SMI (*n* = 28 010)**	**SMI (*n* = 762)**	***P*-value**	**Non-SMI (*n* = 2544)**	**SMI (*n* = 100)**	***P*-value**
			
	***n* (%)**	***n* (%)**		***n* (%)**	***n* (%)**		***n* (%)**	***n* (%)**		***n* (%)**	***n* (%)**	
BP record	1251 (99.4)	44 (97.8)	0.206	4079 (99.3)	94 (96.9)	0.009^b^	27 859 (99.5)	754 (99.0)	0.061	2519 (99.0)	96 (96.0)	0.004^b^
Smoking status record	1257 (99.8)	45 (100.0)	0.789	4099 (99.8)	96 (99.0)	0.133	27 977 (99.9)	759 (99.6)	0.034^a^	2537 (99.7)	97 (97.0)	<0.001^c^
HbA1c record	805 (63.9)	26 (57.8)	0.398	2728 (66.4)	67 (69.1)	0.580	16 468 (58.8)	531 (69.7)	<0.001^c^	1544 (60.7)	69 (69.0)	0.095
Cholesterol record	1206 (95.8)	45 (100.0)	0.160	4017 (97.8)	94 (96.9)	0.576	26 880 (96.0)	734 (96.3)	0.618	2441 (96.0)	94 (94.0)	0.336
BMI record	1187 (94.3)	45 (100.0)	0.099	3849 (93.7)	94 (96.9)	0.193	26 386 (94.2)	743 (97.5)	<0.001^c^	2317 (91.1)	95 (95.0)	0.174
Alcohol record	992 (78.8)	45 (100.0)	0.001^c^	3325 (80.9)	88 (90.7)	0.015^a^	22 637 (80.8)	716 (94.0)	<0.001^c^	1966 (77.3)	92 (92.0)	0.001^c^
eGFR record	1229 (97.6)	44 (97.8)	0.945	3987 (97.0)	94 (96.9)	0.943	26 854 (95.9)	731 (95.9)	0.936	2415 (94.9)	96 (96.0)	0.631
CVD risk factor assessment	236 (18.8)	10 (22.2)	0.558	727 (17.7)	11 (11.3)	0.104	9995 (35.6)	230 (30.2)	0.002^b^	460 (18.1)	14 (14.0)	0.297
TSH record	1140 (90.6)	40 (88.9)	0.709	3619 (88.1)	85 (87.6)	0.893	23 884 (85.3)	677 (88.9)	0.006^b^	2142 (84.2)	86 (86.0)	0.627
CHD comorbidity	569 (45.2)	13 (28.9)	0.031^a^	–	–	–	2590 (9.3)	57 (7.5)	0.096	454 (17.9)	19 (19.0)	0.768
DM comorbidity	428 (34.0)	17 (37.8)	0.599	1294 (31.5)	31 (32.0)	0.922	6837 (24.4)	276 (36.2)	<0.001^c^	647 (25.4)	36 (36.0)	0.018^a^
HYP comorbidity	886 (70.4)	27 (60.0)	0.136	2590 (63.0)	57 (58.8)	0.389	–	–	–	1680 (66.0)	66 (66.0)	0.994

QOF target achievement^d^												
Last BP record within 9 months	–	–	–	–	–	–	18 286 (65.3)	500 (65.6)	0.849	–	–	–
Normal BP (150/90 mmHg) in last 9 months	–	–	–	–	–	–	20 829 (74.4)	557 (73.1)	0.430	1907 (75.0)	67 (67.0)	0.073
Normal BP (150/90 mmHg) in last 15 months	–	–	–	3451 (84.0)	80 (82.5)	0.688	–	–	–	–	–	–
Cholesterol record in last 15 months										1786 (70.2)	69 (69.0)	0.796
Cholesterol <5 mmol/l in last 15 months	–	–	–	2816 (68.5)	58 (59.8)	0.067	–	–	–	1477 (56.9)	52 (52.0)	0.334
Anticoagulant/antiplatelet last 15 months	–	–	–	3002 (73.1)	69 (71.1)	0.667	–	–	–	1460 (61.7)	59 (62.8)	0.840^e^
Quadruple therapy^f^	–	–	–	1530 (51.9)	28 (41.2)	0.082	–	–	–	–	–	–
Beta blocker	879 (69.8)	18 (40.0)	<0.001^c^	2710 (66.0)	53 (54.6)	0.020^b^	–	–	–	–	–	–
ACEI/ARB	1051 (83.5)	28 (62.2)	<0.001^c^	–	–	–	–	–	–	–	–	–

ACEI/ARB = angiotensin-converting enzyme inhibitor/angiotensin receptor blocker. BMI = body mass index. BP = blood pressure. CHD = coronary heart disease. CVD = cardiovascular disease. DM = diabetes mellitus. EGFR = estimated glomerular filtration rate. HbA1c = glycated haemoglobin. HYP = hypertension. MI = myocardial infarction. QOF = Quality and Outcomes Framework. TSH = thyroid stimulating hormone.

a *P* <*0.05.*

b *P*<*0.01.*

c *P*<*0.001.*

d Refers to QOF guidelines 2012/13.^17^

e *If non-haemorrhagic (non-SMI n* = *2366, SMI n* = *94).^2^ If registered with MI (non-SMI n* = *2951, SMI n* = *68). All QOF management guidelines refer to records since registration with outcomes.*

f MI drugs —‘quadruple therapy’ including statin, antiplatelet/anticoagulant, beta blocker, and ACEI/ARB prescription.

### Regression analyses of QOF target achievement

Regression analyses (Table 4) focused on differences in CVD prescribing by SMI status as these differences have previously been identified as a potential contributor to excess cardiovascular mortality among patients with SMI,^12^ and were the key differences identified in Table 3. Associations between SMI status and beta blocker and ACEI/ARB medication among patients with HF remained after accounting for both sociodemographic characteristics and consultation rates. Among patients with CHD, the association between SMI status and beta blocker prescription was accounted for by ethnic group but the shortfall in ACEI/ARB prescribing among CHD patients with SMI remained after adjustments.

**Table 4.** Differences in QOF CVD prescribing targets^a^ by serious mental illness status adjusted for sociodemographic characteristics and primary care consultation frequency

	**Reference (non-SMI)**	**Unadjusted OR (95% CI)**	**Adjusted for sociodemographics OR^b^ (95% CI)**	**Additionally adjusted for consultation rate OR^c^ (95% CI)**
**Beta blocker**				
After CHD	1.00	0.62 (0.41 to 0.93)^d^	0.68 (0.44 to 1.05)	0.66 (0.42 to 1.01)
After HF	1.00	0.29 (0.16 to 0.53)^f^	0.29 (0.15 to 0.55)^f^	0.27 (0.14 to 0.52)^f^

**ACEI/ARB**				
After CHD	1.00	0.59 (0.36 to 0.97)^d^	0.55 (0.33 to 0.94)^d^	0.47 (0.27 to 0.80)^e^
After HF	1.00	0.33 (0.18 to 0.61)^f^	0.34 (0.18 to 0.66)^f^	0.31 (0.16 to 0.60)^f^

**Antiplatelet/anticoagulant**				
After CHD	1.00	0.95 (0.54 to 1.65)	1.04 (0.57 to 1.89)	0.94 (0.51 to 1.73)
After STIA	1.00	1.04 (0.68 to 1.60)	0.99 (0.62 to 1.59)	1.04 (0.64 to 1.69)

**Statin**				
After CHD	1.00	0.76 (0.45 to 1.28)	0.78 (0.45 to 1.36)	0.70 (0.40 to 1.23)

**Quadruple therapy^g^**				
After CHD	1.00	0.65 (0.40 to 1.06)	0.62 (0.37 to 1.04)	0.28 (0.34 to 0.98)^d^

ACEI/ARB = angiotensin-converting enzyme inhibitor/angiotensin receptor blocker. CHD = coronary heart disease. CVD = cardiovascular disease. HF = heart failure. OR = odds ratio. QOF = Quality and Outcomes Framework. SMI = severe mental illness. STIA = stroke/ transient ischaemic attack.

a Refers to QOF guidelines 2012/13.^17^

b Adjusted for age (continuous), sex, ethnic group, and borough-level deprivation.

c Additionally adjusted for mean annual number of primary consultations.

d *P* <*0.05.*

e *P* <*0.01.*

f *P* <*0.001.*

g Quadruple therapy indicated in patients with history of myocardial infarction and includes statin, antiplatelet/anticoagulant, beta blocker, and ACEI/ARB medication.

For analyses examining SMI-subgroups associated with beta blocker and ACEI/ ARB prescribing, CHD and HF were combined because of small numbers (Table 5). After adjustments, prescribing of beta blocker and ACEI/ARB medication among patients with CHD or HF combined was significantly lower for patients with SMI overall (OR 0.48 and 0.42, respectively); and was particularly reduced for patients ever prescribed depot injectable antipsychotic medication (OR 0.22 and 0.32, respectively), those with any indicator of risk (OR 0.25 and 0.22, respectively), those diagnosed with schizophrenia (OR 0.38 and 0.27, respectively), and those with any indicator of SMI severity (OR 0.39 and 0.31, respectively).

**Table 5.** Serious mental illness characteristics associated with beta blocker and ACEI/ARB prescribing among patients with coronary heart disease and heart failure

	**Beta blockers if recorded with CHD or HF (*n* = 3347)**				**ACEI/ARB if recorded with CHD or HF (*n* = 3760)**			

	***n* (%)**	**Unadjusted OR (95% CI)**	**Adjusted for sociodemographics OR^a^ (95% CI)**	**Additionally adjusted for consultation rate OR^b^(95% CI)**	***n* (%)**	**Unadjusted OR (95% CI)**	**Adjusted for sociodemographics OR^a^ (95% CI)**	**Additionally adjusted for consultation rate OR^b^ (95% CI)**
Non-SMI	3279 (68.3)	1.00	1.00	1.00	3677 (76.6)	1.00	1.00	1.00
SMI overall	68 (52.7)	0.52 (0.36 to 0.73)^e^	0.50 (0.35 to 0.73)^e^	0.48 (0.33 to 0.69)^e^	83 (64.3)	0.55 (0.38 to 0.79)^e^	0.49 (0.34 to 0.73)^e^	0.42 (0.28 to 0.62)^e^

**SMI by diagnosis**								
Schizophrenia	30 (50.0)	0.46 (0.28 to 0.77)^d^	0.42 (0.24 to 0.73)^d^	0.38 (0.22 to 0.67)^e^	36 (60.0)	0.46 (0.27 to 0.77)^d^	0.35 (0.20 to 0.60)^e^	0.27 (0.15 to 0.48)^e^
Bipolar affective disorder	8 (40.0)	0.31 (0.13 to 0.76)^c^	0.37 (0.15 to 0.94)^c^	0.35 (0.14 to 0.90)^c^	11 (55.0)	0.37 (0.15 to 0.90)^c^	0.49 (0.18 to 1.26)	0.41 (0.16 to 1.09)
Other non-organic psychoses	8 (61.5)	0.74 (0.24 to 2.27)	0.78 (0.25 to 2.42)	0.75 (0.24 to 2.33)	12 (92.3)	3.66 (0.48 to 28.2)	3.81 (0.49 to 29.4)	3.44 (0.44 to 26.7)

**Depot injectable**								
No	42 (56.8)	0.61 (0.38 to 0.97)^c^	0.58 (0.36 to 0.96)^c^	0.56 (0.34 to 0.92)^c^	48 (64.9)	0.56 (0.35 to 0.91)^c^	0.49 (0.29 to 0.81)^d^	0.43 (0.26 to 0.72)^e^
Yes	11 (36.7)	0.27 (0.13 to 0.57)^e^	0.26 (0.12 to 0.60)^d^	0.22 (0.09 to 0.52)^e^	18 (60.0)	0.46 (0.22 to 0.95)^c^	0.41 (0.18 to 0.91)^c^	0.32 (0.14 to 0.72)^d^

**Typical antipsychotic**								
No	28 (50.9)	0.48 (0.28 to 0.82)^d^	0.50 (0.28 to 0.89)^c^	0.49 (0.27 to 0.86)^c^	34 (61.8)	0.49 (0.29 to 0.85)^c^	0.42 (0.23 to 0.75)^d^	0.37 (0.21 to 0.67)^e^
Yes	25 (51.0)	0.48 (0.27 to 0.85)^c^	0.44 (0.24 to 0.81)^d^	0.39 (0.21 to 0.73)^d^	32 (65.3)	0.57 (0.32 to 1.03)	0.52 (0.28 to 0.97)^c^	0.42 (0.22 to 0.80)^d^

**Atypical antipsychotic**								
No	8 (87.1)	0.62 (0.21 to 1.78)	0.59 (0.20 to 1.71)	0.54 (0.18 to 1.58)	8 (57.1)	0.41 (0.14 to 1.18)	0.41 (0.14 to 1.20)	0.32 (0.10 to 0.96)^c^
Yes	45 (50.0)	0.46 (0.31 to 0.70)^e^	0.45 (0.29 to 0.71)^e^	0.43 (0.27 to 0.67)^e^	58 (64.4)	0.55 (0.36 to 0.86)^d^	0.47 (0.30 to 0.76)^d^	0.41 (0.26 to 0.66)^e^

**Any indicator of severity^f^**								
No	45 (57.0)	0.61 (0.39 to 0.96)^c^	0.56 (0.35 to 0.91)^c^	0.54 (0.33 to 0.87)^c^	56 (70.9)	0.74 (0.46 to 1.21)	0.61 (0.37 to 1.01)	0.52 (0.31 to 0.87)^c^
Yes	23 (46.0)	0.39 (0.23 to 0.69)^e^	0.43 (0.24 to 0.77)^d^	0.39 (0.21 to 0.71)^d^	27 (54.0)	0.36 (0.20 to 0.63)^e^	0.37 (0.20 to 0.66)^e^	0.31 (0.17 to 0.56)^e^

**Any indicator of risk^g^**								
No	54 (59.3)	0.68 (0.44 to 1.03)	0.64 (0.41 to 1.00)	0.61 (0.39 to 0.96)^c^	64 (70.3)	0.72 (0.46 to 1.14)	0.65 (0.40 to 1.04)	0.56 (0.35 to 0.91)^c^
Yes	14 (36.8)	0.27 (0.14 to 0.52)^e^	0.28 (0.14 to 0.57)^e^	0.25 (0.12 to 0.51)^e^	19 (50.0)	0.31 (0.16 to 0.58)^e^	0.27 (0.14 to 0.54)^e^	0.22 (0.11 to 0.44)^e^

ACEI/ARB = angiotensin-converter enzyme inhibitor/angiotensin receptor blocker. CHD = coronary heart disease. HF = heart failure. OR = odds ratio. SMI = severe mental illness.

a Adjusted for age (continuous), sex, ethnic group, borough-level deprivation, and recorded CHD/HF.

b Additionally adjusted for mean annual number of primary consultations.

c *P* <*0.05.*

d *P* <*0.01.*

e *P* <*0.001.*

f Includes any of: ever had an inpatient stay, any record of being treated under the Mental Health Act, any record of difficulty managing their physical health, or any record of an Assertive Outreach/Crisis/A&E episode.

g Includes any of: recorded history of violence, recorded history of non-compliance, and any record of a forensic history.

## DISCUSSION

### Summary

Elevated rates of CVDs were found among patients with SMI; however, there may be underrecording of CVD comorbidities among patients with SMI and with established CVDs. Risk factor recording was high, although significant differences by SMI status were identified. Overall, QOF target achievement was not impaired in patients with SMI but significant consistent associations were found between SMI status and reduced prescribing of ACEI/ARB and beta blocker medication as secondary prevention of CHD and HF. Patients with SMI and with schizophrenia, those identified with any indicator of risk or illness severity, and those ever prescribed depot injectable antipsychotics were least likely to be prescribed ACEI/ARBs and beta blockers.

### Strengths and limitations

This study makes use of a population-based data linkage between primary and secondary care records. It was possible to identify patient and illness-related characteristics associated with recording and treatment of CVDs and to highlight issues warranting further investigation that may best target disparities and reduce inequalities in physical comorbidity and mortality.

The main limitation pertains to the generalisability to other geographical areas; however, the present findings are in line with evidence from national and international research, and it is believed that this study is proof of principle of the utility of data linkage, which could be used elsewhere to corroborate the findings. Although the analyses focus on incentivised QOF targets, it is possible that discrepancies in non-QOF targets may differ.

### Comparison with existing literature

Although patients with SMI were more likely to be recorded with CVDs overall, little evidence was found for elevated rates of CVD comorbid conditions among those with established CVDs. Previous research has found no difference in the pattern of physical health co- and multimorbidities by SMI status and lower than expected rates of certain CVDs among patients with SMI given higher CVD-related mortality.^3^,^19^,^20^,^21^

One of several explanations suggested is that this may be linked to less frequent GP consultations^20^,^21^; however, in this study, elevated consultation rates are reported among patients with SMI overall, and among patients with SMI and with established CVD, in line with previous findings.^22^ Patients with SMI were less likely to have a CVD risk assessment, and although such tools may not be as accurate for the SMI population,^23^,^24^ it is unclear whether this concern or other factors accounted for this observation.

Lower than expected differences were found in the proportion of black Caribbean patients with SMI among those with CHD and STIA. This suggests that either SMI status does not confer an excess risk of these outcomes or that CHD and STIA is less frequently recorded among black Caribbean patients with SMI; for example, because of excess mortality. In line with previous findings,^7^,^14^,^21^,^25^ this study found evidence for reduced prescription of ACEI/ ARB and beta blocker medications for CVD secondary prevention. Underprescribing in CVDs has been linked previously with excess mortality among patients with SMI^7^,^12^,^21^,^25^,^26^ and therefore may contribute to disparities in life expectancies. Reduced ACEI/ARB prescribing in CHD among patients with SMI could partly reflect differences in the effectiveness of these drugs as hypotensive agents among black Caribbean and black African patients.^27^ National Institute for Health and Care Excellence (NICE) HYP guidelines^28^ indicate prescribing of ARBs rather than ACEIs among black patients; however, the associations remained after adjustments for ethnic group and were robust when ACEI and ARB prescriptions were analysed separately. Reduced prescribing is also unlikely to be linked to reduced attendance at primary care as greater consultation frequency was found among patients with SMI, and adjustments strengthened negative associations with prescribing.

There may, however, be reluctance to prescribe certain CVD medications because of concerns about adherence. Adherence may be lower for drugs where the dose has to be up-titrated to maximally tolerated doses as for beta blockers and ACEI/ARBs; these medications require monitoring, and thus adherence to a monitoring regimen to assess for side-effects. Monitoring also involves regular blood tests; such a commitment may be perceived as too demanding for GPs assessing patients with SMI, and/or patients with SMI may be less willing to commit themselves to such monitoring. However, a recent US study assessing adherence in patients with and without schizophrenia found no evidence for reduced adherence to ACEI/ARB medication.^29^ One reason previously suggested for reluctance to prescribe certain cardiovascular medications is the potential for harm in overdose.^14^,^21^ Although research does not support an association between cardiovascular medication and excess suicide,^30^,^31^ practitioners could conceivably have concerns around correct adherence among patients with SMI, for example, leading to accidental overdose.

Further quantitative and qualitative work may usefully further explore these explanations. Qualitative evidence suggests that primary care physicians may view patients with SMI as harder to manage,^31^,^32^ and be less willing to intervene when cardiovascular risk factors are identified.^33^ Further, there may be reluctance among patients with SMI to accept prescriptions because of mistrust or lack of adequate communication between physician and patient.^34^ For patients with greater illness severity, the role of secondary care physicians may be more pertinent in managing physical health.

Lastly, QOF exception rates (for example, because of informed dissent or treatment unsuitability) are higher in patients with SMI,^35^,^36^ potentially inflating QOF achievement. The present analyses did not exclude exception reported patients, however, so the reported achievement rates were not influenced by exception reporting among patients with SMI.

Beta blocker and ACEI/ARB prescription was reduced in patients with SMI with CHD or HF overall, but the reduction was greatest in patients with SMI identified with any indicator of risk, prescription of depot injectable antipsychotics, schizophrenia diagnosis, and any indicator of SMI severity. To the authors’ knowledge, these associations have not been previously investigated; however, Laursen *et al* 25 reported that rates of ‘unnatural’ deaths were elevated among patients with SMI who were not prescribed cardiovascular medication, also indicating an association with illness severity. The subgroups identified as most at risk of underprescribing may be those most likely to be seen as the ‘hardest to treat’ by GPs and those least likely to commit to the monitoring and follow-up as implied before. Further qualitative work should explore these associations among clinicians and patients who have been identified as at risk of underprescribing.

### Implications for practice

These findings deepen the understanding of disparities in morbidity and health care among individuals with SMI and help to build possible explanations for these discrepancies by identifying characteristics of patients with SMI associated with the lowest likelihood of optimal treatment. The results underline the value of closer working between primary and secondary care in improving outcomes for patients with SMI.
